# Takayasu arteritis presenting with massive cerebral ischemic infarction in a 35-year-old woman: a case report

**DOI:** 10.1186/1752-1947-7-179

**Published:** 2013-07-05

**Authors:** Shan Gao, Ruilan Wang

**Affiliations:** 1 Intensive Care Unit, Shanghai First People's Hospital, School of Medicine, Shanghai Jiao Tong University, 650 Xinsongjiang Road, Shanghai 201620, China

## Abstract

**Introduction:**

Takayasu arteritis is a relatively rare type of large-vessel arteritis that primarily affects the aorta and its major branches, the coronary arteries, and the pulmonary arteries. Depending on the different groups of blood vessels involved in the disease process, the clinical presentation of Takayasu arteritis varies. Here we report a case of a woman presenting with a debilitating massive cerebral ischemic infarct that turned out to be a relatively rare first presentation of Takayasu arteritis.

**Case presentation:**

A 35-year-old Chinese woman presented to the Emergency Department with left hemiparesis, pain and numbness of her arms and weak radial pulses. Her laboratory results showed an elevated C-reactive protein and erythrocyte sedimentation rate, and subsequent digital subtraction angiography demonstrated narrowing and occlusion of the major branches of her aortic arch. We report the case of a patient with Takayasu arteritis presenting with a massive cerebral ischemic infarct and review the current literature on this topic.

**Conclusion:**

Takayasu arteritis is a relatively rare disease with various and sometimes devastating clinical manifestations, such as massive cerebral ischemic infarction as in our case. Currently, there are multiple diagnostic tools and treatment options available, and more under investigation. Early, appropriate diagnosis and initiation of proper therapy could avoid further progression and reduce complications of the disease.

## Introduction

Takayasu arteritis is a relatively rare type of large-vessel arteritis that primarily affects the aorta and its major branches, the coronary arteries, and the pulmonary arteries. Here we report a case of a woman presenting with debilitating massive cerebral ischemic infarction that turned out to be Takayasu arteritis.

## Case presentation

A 35-year-old Chinese woman presented to the Emergency Department (ED) after a fall 12 hours previously and with an inability to move her left limbs since then. She complained of feeling pain and numbness in her arms for the past month, as well as progressive weakness in her legs. She had never been pregnant, had no significant past medical or surgical history, nor was she on any medications such as oral contraceptive pills. There was no family history of early onset cerebral vascular accident. She denied the use of alcohol, tobacco products or recreational drugs. On physical examination, she was conscious and irritable, her heart rate was 88 beats per minute (bpm), blood pressure (BP) was recorded as 140/90mmHg upon ED presentation. She had no fever or respiratory distress. She was unable to open her right eye and had a right-sided preferred gaze with slight right-sided facial droop. There was obvious left-sided hemiparesis with a positive Babinski sign, and the skin of her left side felt cold. The radial pulse was weak on both sides. The rest of her neurological, systemic, and general physical examinations was unremarkable.

**Figure 1:**
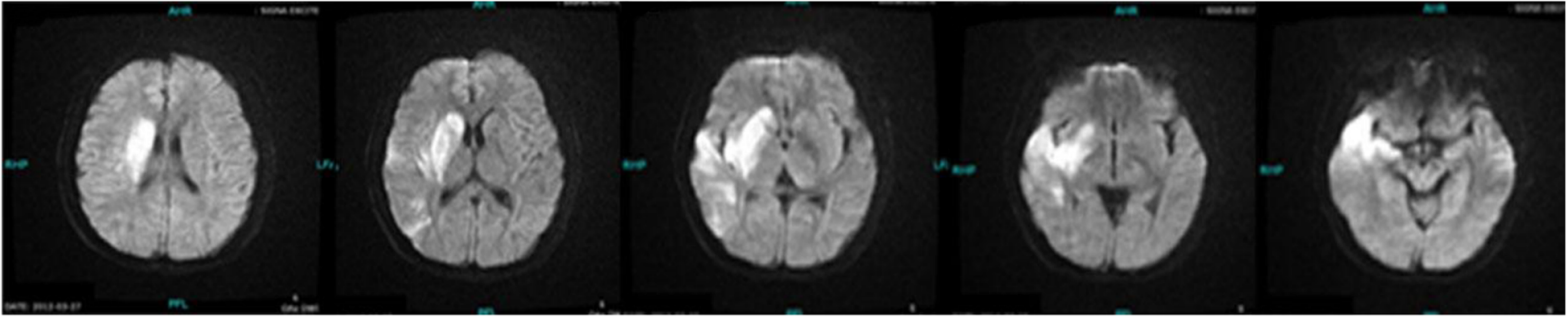
Brain magnetic resonance imaging (MRI) scan showing massive acute ischemic infarct.

**Figure 2:**
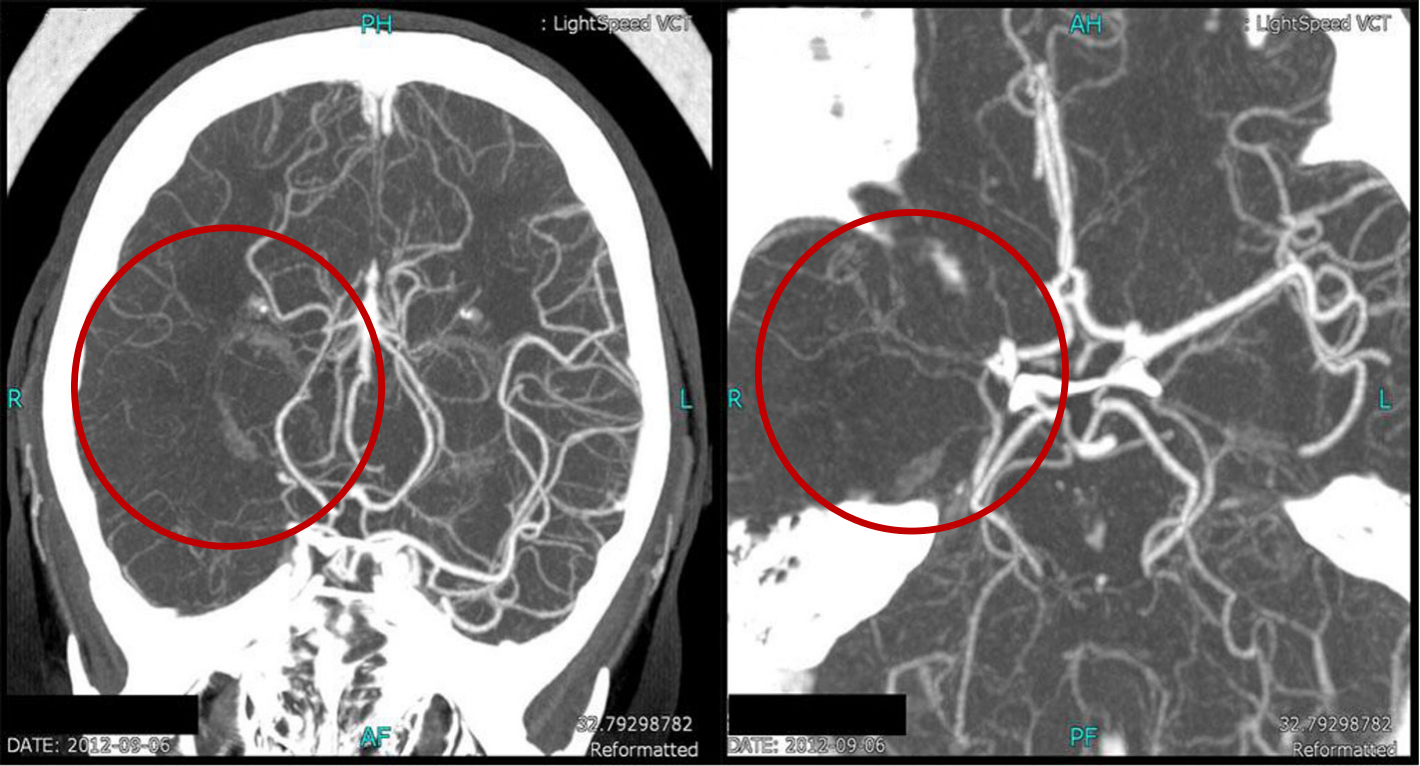
Head computed tomographic angiography (CTA) showing absence of the middle cerebral artery (MCA) on the right side (red circle).

**Figure 3:**
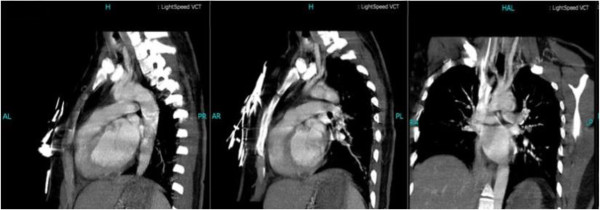
Thoracic and abdominal computed tomographic angiography (CTA) was negative.

**Figure 4:**
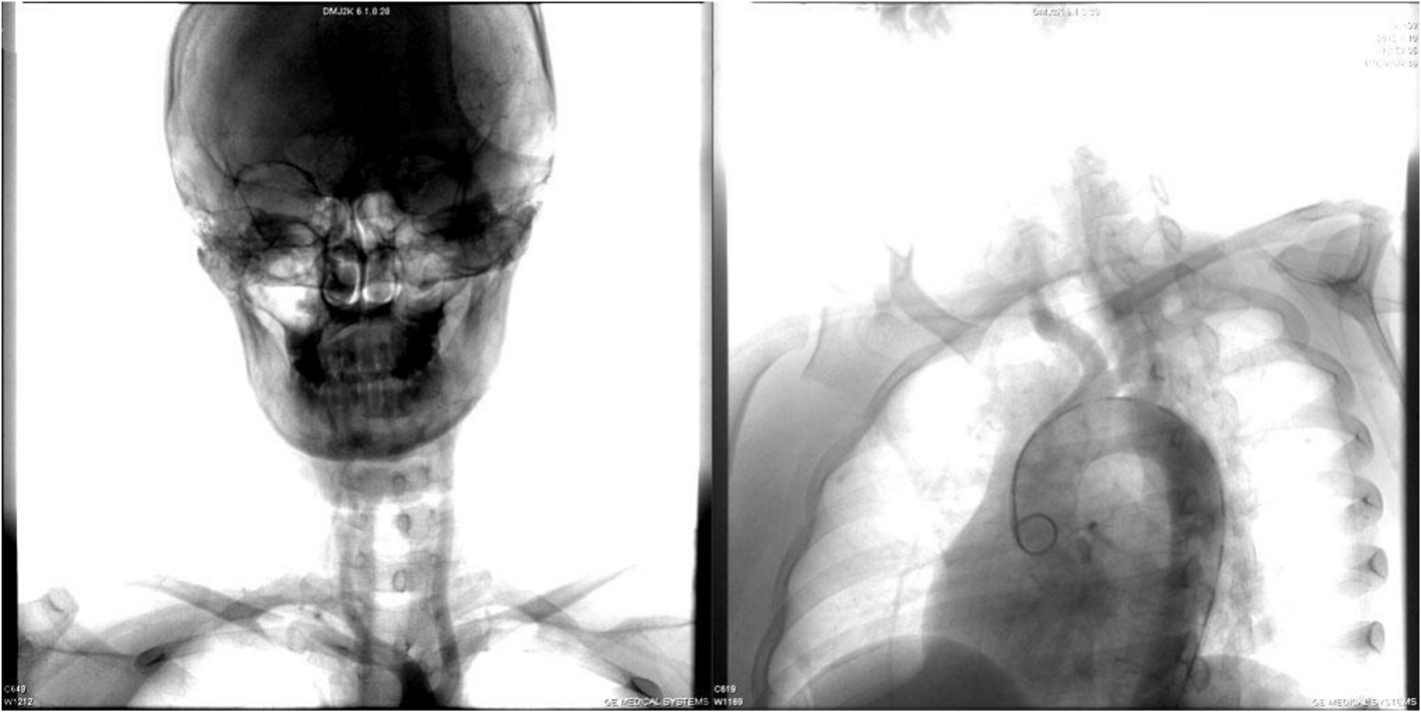
Digital subtraction angiography (DSA) showing occlusion of the right subclavian artery and narrowing of the right internal carotid artery.

Her laboratory test results showed white blood cells (WBCs) of 13.9x109/L, hemoglobin (Hb) of 70.2g/L, and platelets (PLTs) of 288x109/L. Her basic metabolic panel was unremarkable. Her coagulation test was only significant for elevated D-dimer of 1.08mg/L, which subsequently dropped to 0.96mg/L on the third day of her hospital stay, with partial thromboplastin time (PT) and activated partial thromboplastin time (APTT) both within normal limits. The lipid panel showed slightly elevated cholesterol of 5.83mmol/L (2.85 to 5.2mmol/L), triglyceride of 1.79mmol/l (0.5 to 1.7mmol/L) and lipoprotein A of 373 (0 to 300). Her inflammatory biomarkers such as C-reactive protein (CRP) and erythrocyte sedimentation rate (ESR) were both elevated, with 26.0mg/L (0 to 8.0mmol/L) and 25mm/h (0 to 20mm/h) respectively. Rapid plasma reagin (RPR)/treponema pallidum particle agglutination (TPPA) tests were negative. Autoantibody tests showed positive for antinuclear antibody only, with a titer of 1:100, while the antiphosphlipid antibody test was negative. Her blood glucose, liver and renal functions were all normal. Her electrocardiogram (EKG) and transthoracic echocardiogram were both negative. A cerebral magnetic resonance imaging (MRI) scan showed acute ischemic infarction of the area supplied by the right middle cerebral artery (Figure 1), which was proven to be occluded by later cerebral computed tomographic angiography (CTA) (Figure 2). Thoracic and abdominal CTA was negative (Figure 3). A Doppler sonogram of the vessels in the neck, upper and lower limbs was unable to find both axillary arteries or the left subclavian artery. It did show decreased blood flow in the arteries of her right arm, with a narrowed right subclavian artery. The arteries distal to the brachial artery were visible on both sides, with a greater blood flow on the left. Subsequent digital subtraction angiography (DSA) confirmed the incomplete occlusion of her right subclavian artery and narrowing of her right internal carotid artery (Figure 4).

The diagnosis of Takayasu arteritis type I was made according to the revised Ishikawa diagnostic criteria [1] and the angiographic classification of Takayasu arteritis put forward at the Takayasu Conference 1994 [2]. Subsequently, the recommended treatment of methylprednisolone was started with an initial dose of 40mg per day. As the antinuclear antibody test was positive, autoimmune diseases such as systemic lupus erythematosus (SLE) could not be excluded, which in the light of the existing thrombosis, could be related to a hypercoagulation state. Thus a small dose of low-molecular-weight heprin (LMWH) was started along with the steroid.

Twenty days after admission, the clinical condition of the patient was stable, and she was subsequently discharged.

## Discussion

The most common types and clinical presentations of Takayasu arteritis vary in different ethnic groups [3]. A retrospective study of 125 patients with Takayasu arteritis conducted by Cong *et al.* suggested that the most common type in the Chinese population is type I [4], which our patient fell into. However, according to the same study, the most common clinical manifestation of Takayasu arteritis in China is pulse deficit (71.2%) followed by hypertension (65.6%), and the common neurological findings tend to be dizziness (50.4%) and visual disturbance (33.6%) [5]. The presentation of our patient is relatively rare and devastating, thus raising the question of the etiology of her ischemic stroke. Based on her clinical manifestation and MRI scan, this cerebral vascular accident seemed to be embolic in nature. There are several possible explanations of the source of the embolus. A cardiac source was probably excluded by the negative transthoracic echocardiogram, although a transesophageal echocardiogram with bubble study was not done. There was a similar case reported by Kato *et al.* who suggested a stump of the occluded common carotid artery leading to turbulence of blood flow as a possible source of embolization [6]. We also suspected the coexistence of a hypercoagulation state in our patient. In our review of the existing literature, some studies suggested that Takayasu arteritis itself could cause an overactivation of the coagulation system via different pathways [5,7,8]. In the meanwhile, there are reported cases of Takayasu arteritis where the antibodies of antiphosphlipid syndrome were also positive [9-12], which were related to more severe clinical courses. In our case, the antiphosphlipid antibody itself was negative, but there are other entities of antiphosphlipid syndrome that we failed to test for due to an availability issue [13]. The positive antinuclear antibody result also raised suspicion of autoimmune disorders such as SLE, which could cause hypercoagulation as well. Thus, the empiric anticoagulation therapy was warranted.

Several imaging tools were utilized in our case. Among these technologies, CTA is considered to be more sensitive to large-vessel lesions rather than relatively small vessels such as the distal portions of aortic branches [14,15] and has a lesser resolution compared to ultrasound [16]. But overall, CTA and Doppler both have great sensitivity and specificity in terms of the diagnosis of Takayasu arteritis, and they are able to show early changes in the vessel wall that DSA fails to illustrate. The method we did not use on this patient is positron emission tomography/computed tomography (PET/CT) due to issues of cost and radiation exposure. As a functional imaging modality, PET/CT is able to pick up sites of active inflammation, which makes it a useful tool in detecting the early-stage vascular lesions and monitoring the activity of the disease [17]. In regard to treatment options of Takayasu arteritis, corticosteroid remains the first-line medical therapy in the active disease process. In our patient, since the clinical and laboratory manifestations were both improving with steroid use only, other immunosuppressants were not used. Surgical interventions such as angioplasty and vascular reconstruction are also recommended for severe stenosis or occlusion of critical arteries, between which surgical bypass is considered to have superior potency but more serious early postoperative complications as well [18]. While there is no commonly accepted criteria for the indications of surgical intervention in Takayasu arteritis, cerebrovascular ischemia, as presented in this case, is considered a strong indication [19,20]. However, the nature of Takayasu arteritis as an inflammatory disease raises the failure and complication rate of surgical interventions [21] and surgery is contraindicated in the acute phase of the disease. So this treatment option will be discussed during the subsequent follow-ups of our patient.

As seen in our patient, Takayasu arteritis can cause devastating damage to the overall health condition and quality of life, or even be life-threatening sometimes. So it would be beneficial to diagnose the disease at an early stage and monitor its activity and progression, which remains a challenge that physicians and experts all over the world have been working on, due to such a wide range of clinical manifestations this disease can present with. In our patient, initial physical examination demonstrated reduced radial pulses on both sides, which raised suspicion of Takayasu arteritis. According to a recent study, individual physical examination findings had poor sensitivity and good to excellent specificity [22]. While various imaging techniques and blood levels of CRP and ESR remain the most useful tools and the gold standard in diagnosing and monitoring the progress of the disease, several biomarkers have been suggested by recent studies as well, which include IL-6 [23], matrix metalloproteinase (MMP)-2, MMP-3, MMP-9 and pentraxin 3 (PTX3) [24]. But all of these biomarkers are still under investigation and none of them has showed a clear-cut correlation with the disease activity. As a result, the historical Ishikawa clinical classification of Takayasu arteritis [25] is still widely used to determine prognosis and treatment plan. If diagnosed at an early stage, the disease could be controlled with the standard therapies discussed earlier, as well as surgical intervention if critical vessels are involved, so that debilitating and irreversible complications such as massive cerebral ischemic infarction may not ensue.

## Conclusion

Takayasu arteritis is a relatively rare disease with various and sometimes devastating clinical manifestations, such as the massive cerebral ischemic infarct in our case. Currently, there are multiple diagnostic tools and treatment options available, and more under investigation. Early, appropriate diagnosis and initiation of proper therapy could avoid further progression and reduce complications of the disease.

## Consent

Written informed consent was obtained from the patient for publication of this case report and any accompanying images. A copy of the written consent is available for review by the Editor-in-Chief of this journal.

## Competing interests

The authors declare that they have no competing interests.

## Authors’ contributions

SG drafted the manuscript and finished the data collection. RW developed the idea for the case report and revised the paper. Both authors read and approved the final manuscript.
